# The Immunobiogram, a Novel *In Vitro* Assay to Evaluate Treatment Resistance in Patients Receiving Immunosuppressive Therapy

**DOI:** 10.3389/fimmu.2020.618202

**Published:** 2021-01-25

**Authors:** Jose Maria Portoles, Carlos Jimenez, Dario Janeiro, Maria O. Lopez-Oliva, Alvaro Ortega-Carrion, Daniel Blanquez, Luis Arribas, Carlos Gomez, Teresa Diez, Julio Pascual, Isabel Portero

**Affiliations:** ^1^ Department of Nephrology, Hospital Universitario Puerta de Hierro, Madrid, Spain; ^2^ Research net REDInREN 016/0009, Instituto Salud Carlos III, Madrid, Spain; ^3^ Department of Nephrology, Hospital La Paz, Madrid, Spain; ^4^ R+D Department, BIOHOPE Scientific SL, Madrid, Spain; ^5^ Adamas Engineering Consulting, Madrid, Spain; ^6^ Department of Nephrology, Hospital del Mar, Institut Mar for Medical Research, Barcelona, Spain

**Keywords:** cellular pharmacodynamics, immune cell assay, immunosuppressive therapy monitoring, transplant rejection, personalized medicine

## Abstract

Immunosuppressive drugs are widely used to treat several autoimmune disorders and prevent rejection after organ transplantation. However, intra-individual variations in the pharmacological response to immunosuppressive therapy critically influence its efficacy, often resulting in poor treatment responses and serious side effects. Effective diagnostic tools that help clinicians to tailor immunosuppressive therapy to the needs and immunological profile of the individual patient thus constitute a major unmet clinical need. *In vitro* assays that measure immune cell responses to immunosuppressive drugs constitute a promising approach to individualized immunosuppressive therapy. Here, we present the Immunobiogram, a functional pharmacodynamic immune cell-based assay for simultaneous quantitative measurement of a patient’s immune response to a battery of immunosuppressive drugs. Peripheral blood mononuclear cells collected from patients are immunologically stimulated to induce activation and proliferation and embedded in a hydrogel mixture in which they are exposed to a concentration gradient of the immunosuppressants of interest. Analysis of samples from kidney transplant patients using this procedure revealed an association between the sensitivity of individual patients to the immunosuppressive regimen and their immunological risk of transplant rejection. Incorporation of the Immunobiogram assay into clinical settings could greatly facilitate personalized optimization and monitoring of immunosuppressive therapy, and study of the mechanisms underlying resistance to immunosuppressants.

## Introduction

Immunosuppressive (IS) medications that control and/or modulate the immune response are the treatment of choice for many inflammatory pathologies of autoimmune (1) and non-autoimmune origin, and are essential to prevent immunological graft rejection in transplant patients (2). Their use in clinical practice has increased patient survival and quality of life and allowed for significant improvements in graft survival in transplant recipients (3). The objective of immunosuppressive treatment is to restore the equilibrium of the immune system and/or the patient’s inflammatory state, thereby facilitating the development of physiological tissue repair mechanisms. These beneficial effects are a consequence of partial silencing of the patient’s immune system, which is essential for life (4). Continued use of these drugs can therefore be deleterious, increasing the risk of opportunistic infections, comorbid conditions, or tumors (5, 6).

Current clinical practice avails of a broad range of immunosuppressive drugs for which the underlying mechanisms and specific therapeutic targets are well documented. However, the effects of these drugs are pleiotropic; they target the immune system at multiple levels, and alter the function and activity of a range of immune cell types, resulting in so-called off-target effects that have unwanted immunological consequences, including leukopenia, myelosuppression and an increased incidence of infections and malignancies. The adverse effects of long-term immunosuppressant treatment are not limited to the immune system, and include obesity, diabetes mellitus, hypertriglyceridemia, hepatotoxicity, and pancreatitis (7). Typically, doctors begin treatment with a first-line medication, subsequently switching to a second-line medication if the patient’s clinical condition fails to improve or even worsens. This trial and error approach entails great risk to patients with any one of a large number of diseases, and to organ transplant recipients (8). Indeed, adverse effects of immunosuppressive drugs and excess immunosuppression account for a large proportion of the mortality associated with immune-based inflammatory diseases (9, 10).

Maintenance immunosuppressive therapy directly or indirectly inhibits cell proliferation, leukocyte migration, and the activity of immune cells, thus preventing their migration to the target tissue in large numbers to perform their effector functions (11). Crucially however, not all patients respond equally to a given immunosuppressant drug. This is due to individual differences in pharmacokinetic and pharmacodynamic responses to drugs, as well as the complexity of their targets: The immune system comprises a vast network of humoral and cellular interactions regulated at multiple levels (12). Genotypic, epigenetic, and pharmacogenetic factors thus influence the cellular response to immunosuppressants and contribute to differences in resistance to immunosuppressive therapy. Each patient has a specific immunological profile that, at a certain point during the treatment, will strongly influence their response to any immunosuppressive treatment (13, 14).

Current monitoring approaches of immunosuppressive therapy are mainly based on clinical monitoring and measurement of immunosuppressive drug levels. These pharmacokinetic data serve as surrogate indicators of the true extent of immunosuppression in the patient but provide no information on the drug’s effects on discrete subsets of immune cells. Moreover, the marked discrepancies between these pharmacokinetic parameters and the clinical efficacy of immunosuppressants in individual patients are well documented (15–18). An alternative approach is to assess the effects of immunosuppressant therapy on specific targets (e.g., on enzyme activity or T-cell subsets) *in vitro*, thereby obtaining a pharmacodynamic readout of immunosuppressant efficacy. Measuring the cellular pharmacodynamics of immunosuppressive drugs has proven as an efficient strategy to predict the clinical efficacy of drugs in many immunological disorders and organ transplantations (19).

In patients that require continuous immunosuppressive therapy, treatment selection could be facilitated by objective and personalized pre-treatment assessment of the efficacy of a range of immunosuppressants based on a specific immunological endpoint. Because a patient’s immune status can vary in parallel with their underlying clinical condition (20) and as a consequence of continuous exposure to immunosuppressants (21), such testing would need to be performed at multiple time points to obtain a series of discrete snapshots of the patient’s immune response. These data could in turn be combined with other prognostic or efficacy biomarkers (22). Such a tool could facilitate selection of the most appropriate immunosuppressant for a given patient, as well as tailored adjustment of the dose administered, thereby improving quality of life and treatment response while minimizing unwanted side effects.

In this paper, we describe the development and initial validation of a novel *in vitro* assay called the Immunobiogram^®^ (IMBG). This functional pharmacodynamic immune-cell-based assay enables the simultaneous quantitative measurement of a patient’s immune response to a battery of immunosuppressive drugs. PBMCs are extracted from the patient’s blood sample and are immunologically stimulated to induce their activation and proliferation. These activated PBMCs are embedded in a hydrogel substrate, which is added to segregated channels in the immunobiogram plate, and each channel is exposed to a concentration gradient of a different immunosuppressive drug. PBMC activation and proliferation across the concentration gradient are measured using a resazurin-based assay, providing a read-out of the immune cell response to each immunosuppressant. To evaluate the feasibility of the immunobiogram, we used PBMCs from healthy blood donors. We next tested the immunobiogram using samples from kidney transplant (KT) patients, who are typically prescribed immunosuppressants to prevent graft rejection. We evaluated each patient’s sensitivity to the immunosuppressive drugs they were prescribed at the time of testing and observed an association between the resulting sensitivity profile and the patient’s risk of rejection. Our findings validate the use of this novel methodology for individualized *in vitro* evaluation of immune cell responses to immunosuppressive drugs, and underscore this assay’s clinical potential for the personalized management and monitoring of patients undergoing immunosuppressive therapy.

## Materials and Methods

### Study Design

The initial study performed with samples from healthy blood donors and the subsequent cross-sectional observational study with samples from KT patients were both conducted in accordance with Spanish Biomedical Research Law 14/2007, of July 3.

Blood samples from 34 apparently healthy blood donors were obtained from the Hemotherapy and Blood Donation Centre of Castilla-León (female, 18% (n = 6); male, 82% (n = 28); mean [range] age, 46.5 [21–59] years. None of the donors were receiving any immunosuppressive treatment at the moment of extraction.

Blood samples from KT recipients were obtained from two major university hospitals (Hospital La Paz and Hospital Puerta de Hierro, Madrid, Spain). The inclusion criteria for KT patients were as follows: men and women aged ≥18 years who had undergone renal transplant at least 1 year before inclusion.

KT recipients were divided into three different groups according to immunological risk (low, intermediate, and high). Patients with a high immunological risk profile were those that required increased immunosuppression, usually due to the presence of clinical or immunological signs related with chronic graft rejection. Specifically, patients assigned to this group were those that fulfilled any of the following criteria: 1) progressive deterioration of renal function during the preceding year, with proteinuria (albumin/Cr urine ratio ≥500 mg/g) and post-transplantation levels of anti-HLA antibodies (Ab) >2000 U; 2) episodes of acute cellular and/or humoral rejection; 3) presence of post-transplantation anti-HLA Ab alone, provided that the Abs are donor specific or that the patient expressed anti-HLA against more than one HLA antigen (Ag) before transplantation. Patients with an intermediate immunological risk profile were those who maintained good graft function with conventional immunosuppression as indicated in the KDIGO guidelines (23). Patients in the low immunological risk (LR) group were those for whom a decrease in the corresponding immunosuppression regimen could be reasonably proposed owing to the development of a certain degree of graft tolerance. Specifically, these patients were undergoing immunosuppression as monotherapy and/or as double therapy at reduced doses (but not triple therapy); had stable kidney function without proteinuria for more than 1 year; had experienced no episodes of acute or chronic rejection; and tested negative for anti-Ag antibodies of the HLA system as determined by Luminex.

The exclusion criteria were as follows: lack of informed consent; active systemic infections that required antimicrobial treatment; HIV, HBV, HCV infection, or other severe infectious diseases that prevent blood sample processing in a conventional laboratory; active immune-based disease with acute outbreaks in the past 12 months, despite immunosuppressive treatment; double transplant (renal + another organ).

The following data on the patients’ clinical and immunological history were collected: sociodemographic variables; medical history including diagnosis of native renal disease; a complete history of the transplantation procedure (donor and patient); time since transplantation; cause; immunological history before and after transplantation; relevant clinical outcomes related to the transplant (mainly rejection episodes); presence of dsDNA and biopsies; and data on current immunosuppressive therapy and other concomitant treatments.

In total, blood samples were acquired from 66 KT patients who fulfilled the inclusion criteria. Of these 66 patients, the IMBG results obtained for six patients were deemed invalid (due to plate reading errors or sample contamination) and were excluded from our analyses. The remaining 60 patients were assigned to following categories: high immunological risk, n = 19; intermediate immunological risk, n = 20; low immunological risk, n = 21. Clinical, immunological, and sociodemographic characteristics of all patients are shown in **Table 1**.

**Table 1:** Demographics and presenting features of the study cohort.

	Low immunological risk N=21	Intermediate immunological risk N=20	High immunological risk N=19
**Recipient characteristics**			
**Age of recipient in years, mean (CI95%)**	63 (56.4–69.6)	57.2 (52.1–62.3)	48.1 (40.4–55.8)
**Male recipients, N (%)**	11 (52%)	13 (65%)	12 (63%)
**Years since last kidney transplantation, mean (CI95%)**	21.50 (18.1–24.9)	6.35 (4.65–8.06)	4.95 (3.58–6.33)
**Previous transplantation, N (%)**	0 (0%)	2 (10%)	11 (58%)
**History of previous acute rejection episodes, N (%)**	0 (0%)	0 (0%)	13 (68%)
**Pre-transplant number of HLA mismatches, mean (CI95%)**	3.3 (2.75–3.85)	4.05 (3.33–4.77)	4.37 (3.72–5.02)
**Pre-transplant number of HLA mismatches >3, N (%)**	14 (67%)	16 (80%)	18 (95%)
**Post-transplant de novo donor-specific antibodies, N (%)**	0 (0%)	0 (0%)	17 (89%)
**Elective biopsy, N (%)**	0 (0%)	1 (5%)	14 (74%)
**Abnormal biopsy, N (%)**	0 (0%)	1 (5%)	13 (68%)
**Biopsy with graft rejection findings, N (%)**	0 (0%)	0 (0%)	13 (68%)
**Donor characteristics**			
**Age of donor in years, mean (CI95%)**	37.5 (28.7–46.4)	51 (45.4–56.5)	44.9 (39.3–50.6)
**Transplant from deceased donor, N (%)**	19 (90%)	17 (85%)	17 (89%)
**Kidney function and other parameters**			
**Mean serum creatinine level, mg/dl (CI95%)**	1 (0.88–1.13)	1.34 (1.12–1.55)	1.77 (1.35–2.19)
**Serum creatinine level >1.5 mg/dl, N (%)**	1 (4%)	5(25%)	13 (68%)
**Proteinuria >500 mg/d, N (%)**	9 (42%)	3 (15%)	8 (42%)
**eGFR (ml/min/1.73 m2) <50, N (%)**	2 (9.5%)	7 (35%)	12 (63%)
**Hemoglobin, mg/dl, mean (CI95%)**	13.9 (13.2–14.7)	13.9 (13.4–14.4)	12.2 (11.2–12.2)
**Naive B lymphocytes/total B lymphocytes >65%, N (%)**	7 (33%)	4 (20%)	11 (58%)
**Neutrophils x 10^3^/mm^3^, mean (CI95%)**	3.63 (2.81–4.44)	4.66 (3.68–5.63)	4.12 (3.50–4.65)
**Neutrophilia >60%, N (%)**	9 (43%)	13 (65%)	13(68%)
**Immunosuppressive treatment**			
**Induction with Thymoglobulin, N (%)**	0%	6 (30%)	15 (79%)
**Treatment with MMF, N (%)**	4 (2%)	16 (80%)	17 (90%)
**Treatment with TAC, N (%)**	2 (9%)	16 (80%)	18 (95%)
**Treatment with CSA, N (%)**	4 (19%)	1 (5%)	1(5%)
**Treatment with an mTOR inhibitor (SIR or EVE), N (%)**	13 (62%)	5 (25%)	1(5%)
**Treatment with corticosteroids, N (%)**	13 (62%)	15 (75%)	19(100%)
**Treatment with AZA, N (%)**	5 (24%)	0 (0%)	0 (0%)
**Number of IS, mean (CI95%)**	1.86 (1.69–2.02)	2.65 (2.42–2.88)	2.95 (2.84–3.06)
**Treatment with 1 IMS, N (%)**	3 (14%)	0 (%)	0 (%)
**Treatment with 2 IMS, N (%)**	18 (86%)	6 (30%)	1 (5%)
**Treatment with 3 IMS, N (%)**	0 (0%)	14 (70%)	17 (89%)
**Treatment with 4 IMS, N (%)**	0 (0%)	0 (0%)	1 (5%)
**Treatment with Mycophenolic+Tacrolimus+Corticosteroids (%)**	0 (0%)	7 (35%)	16 (84%)

The study was approved by the ethical committees of the participating centers, and all patients provided informed consent before participating in the study.

### Sample Collection and Processing

Blood samples (healthy blood donors, 10 ml; KT recipients, 30 ml) were collected by venipuncture, stored in lithium heparin tubes, transported by courier and received 1 day after extraction.

Peripheral blood mononuclear cells (PBMCs) were isolated by density gradient centrifugation using tubes preloaded with Pancoll^®^ (Pan Biotech P04-60125). Isolated PBMCs were then frozen in liquid nitrogen for at least 3 weeks before processing. PBMCs (mainly T-cells) were activated by incubation for 4 days in a standard incubator (37°C, 5% CO_2_) in X-VIVO medium (LONZA cat: BE02-054Q) in the presence of agonistic antibodies (Dynabeads Human T-activator CD3/CD28; Thermo Fisher, cat: 111.32D) at a Dynabead : PBMC ratio of 1:10. CD3 and CD28 exert an activation effect equivalent to that resulting from binding of the physiological membrane ligands MHC (major histocompatibility complex) and CD80/86, respectively (24) (Dynabeads Human T-activator CD3-CD28 technical file). PBMCs cultured in the absence of Dynabeads were used as a negative control.

### Hydrogel Preparation

The hydrogel used for the IMBG consists of X-VIVO medium and a polymer that is compatible with viable 3-dimensional growth of PBMCs and capable of producing a viscoelastic solid-like material.

Methylcellulose was prepared by mixing 45 ml of stock methylcellulose (ClonaCell Flex; Stemcell Technologies, Cat: 03818) with 45 ml of X-VIVO medium and 5 ml of PBS 10X. The mixture was homogenized by vortexing and left on ice for at least 30 mins until disappearance of all bubbles in the solution. Hydrogel was prepared by mixing the aforementioned methylcellulose solution with collagen copolymer (0.5% PureCol^®^ EZ Gel solution) at 4°C at a ratio of 7:3. The volume of hydrogel required was 1 ml per IMBG channel. The final volume of hydrogel prepared varied depending on the number of channels to be loaded per IMBG plate. Hydrogel used for negative controls, to which non-activated cells were added, was prepared separately.

### Immunosuppressant Delivery

Autoclaved paper discs (Divers/Dutcsher Cat: 074074) measuring 6 mm in diameter were used to deliver immunosuppressant drugs to each channel in the IMBG plate. Immunosuppressant drugs were dissolved in pure ethanol (Sigma Aldrich) and stored as stock solutions at -20°C until use. On the day of the assay, in sterile conditions discs were loaded with 15 µl of ethanol containing varying amounts of the immunosuppressant of choice (sirolimus: 7.5 µg; everolimus and tacrolimus: 10 µg; azathioprine: 25 µg; mycophenolic acid, cyclosporine A, and methyl prednisolone: 100 µg each). Discs were incubated for 1 h at 37°C or 3 h at RT to allow evaporation of the ethanol and then placed at the end of the hydrogel+PBMC-loaded channel in the IMBG plate.

### Immunobiogram Assay

The IMBG plate is designed to accommodate two control conditions (C+, positive control [Dynabead-stimulated PBMCs]; C−, negative control [unstimulated PBMCs]), and the following seven immunosuppressant conditions (all tested on Dynabead-stimulated PBMCs): mycophenolic acid (MPA; Sigma, Cat. M3536-50MG); cyclosporine A (CSA; Sigma, Cat. 30024-25MG); tacrolimus (TAC; Sigma, Cat. F4679-5MG); methyl prednisolone (MTP; Sigma, Cat. M3781-25MG); sirolimus (SIR; Sigma, Cat. 37094-10MG); everolimus (EVE; Eurodiagnostic, Cat. HY-10218); azathioprine (AZA; Sigma, Cat. PHR1282-1G).

**Figure 1:**
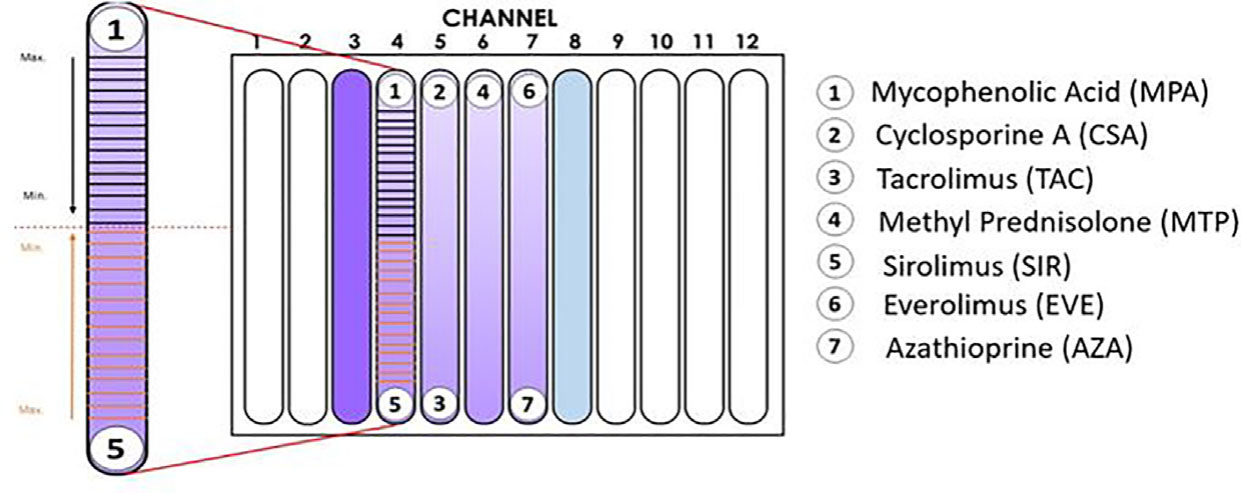
Configuration of the Immunobiogram plate. Cartoon illustrating the location of the immunosuppressant (IMS) discs in the IMBG plate. No discs were placed in channels 3 and 8 (positive and negative controls, respectively). In channels 4–7, activated PBMCs were exposed to concentration gradients of the immunosuppressants indicated on the right-hand side of the cartoon. In the magnified image of the channel on the left-hand side, the horizontal lines indicate the 15 points at which fluorescence readings were taken along the concentration gradients of mycophenolic acid and sirolimus, respectively. Arrows indicate the direction of the concentration gradient, from maximum (closest to each disc) to minimum (at the mid-point of the channel, indicated by dashed red line).

X-VIVO medium containing PBMCs was added to the previously prepared hydrogel solution at a ratio of 1:20 v/v. The number of cells (stimulated or unstimulated PBMCs) added to the corresponding hydrogel container was sufficient to achieve a final concentration of 500,000 cells/ml in the channel. The hydrogel+PBMC mixture and hydrogel was vortexed and maintained at 4°C in a horizontal position until all bubbles had disappeared. Next, the hydrogel + PBMC mixture was added to each channel in the IMBG plate and allowed to sit for 90 min at 37°C. For each assay, the IMBG plate was incubated for approximately 15 h at 37°C and 5% CO_2_ after placement of the discs loaded with the immunosuppressants of interest. No discs were placed in either of the two control channels (positive and negative controls). All experiments were conducted using the configuration shown in **Figure 1**.

Spontaneous diffusion of a substance through hydrogel depends on several parameters, including the characteristics of the solute (e.g., concentration, molecular size, and solubility, polarity) and of the hydrogel network (e.g., pore size, chemical monomer structure, crosslink degree, and mesh size), temperature, pH, and the presence of ions (25). Upon placement of the immunosuppressant disc at one end of a channel on the IMBG plate, the immunosuppressant contained in the disc diffuses passively through the hydrogel in the channel, producing a stable and quantifiable drug concentration gradient (26, 27). This drug concentration gradient exerts a dose-dependent inhibitory effect on the activation and proliferation capacity of the PBMCs embedded in the hydrogel.

After incubation of the IMBG plate, resazurin solution (Presto Blue; Thermo Fisher Cat. A13261), which is used as an oxidation-reduction indicator in cell viability assays, was diluted in X-VIVO medium at a ratio of 1:2 and added with a pipette at a ratio of 1:10 relative to the final volume of hydrogel in each channel. Plates were then incubated for 3 h (37°C, 5% CO_2_) before fluorimetry reading. PBMC fluorescence was measured using a Spark^®^ multimode microplate reader (Tecan) in fluorometric mode at 535/610 nm em/ex. For each of the immunosuppressants tested, fluorescence readings were taken at 15 fixed locations along the channel (see **Figure 1**).

### Flow Cytometry Assay

Peripheral blood mononuclear cells (PBMCs) from healthy donors were isolated by density gradient centrifugation using tubes preloaded with Pancoll^®^ (Pan Biotech P04-60125). Isolated PBMCs were then frozen in liquid nitrogen for at least 3 weeks before processing.

For the activated condition PBMCs (mainly T-cells) were activated by incubation for 4 days in a standard incubator (37°C, 5% CO_2_) in X-VIVO medium (LONZA cat: BE02-054Q) in the presence of agonistic antibodies (Dynabeads Human T-activator CD3/CD28). For the non-activated condition, cells from the same donor were defrosted and maintained in cell culture for 2 days before the assay in X-VIVO medium. Cell activation was evaluated using anti-CD69 and anti-CD25 antibodies (data not shown).

For analyses, 200,000 cells (PBMCs) were used for each condition. Cells in X-VIVO medium were arranged in cytometry tubes in a final volume of 200 µL. Resazurin solution was prepared from resazurin sodium salt (Invitrogen Cat. R12204). Prior to the test the resazurin was resuspended in PBS at a concentration of 440 µM. Once dissolved, the resazurin was added at a dilution of 1/10 (v/v) and incubated at 37°C and 5% CO_2_ for 1 h before reading.

Resazurin-dyed PBMCs were read on a DxFLEX flow cytometer (Beckman Coulter) with a 580-nm emission filter (phycoerythrin). The selection of viable lymphocytes and subsequent analysis of mean fluorescence intensity (MFI) was carried out using Cytexpert software (version 2.0.1.89). Statistical analyses of individual experiments and correlation analyses were performed using GraphPad Prism 8 (version 8.4.2).

### Validation of the Fluorescence Reading Method

After selecting the type of reagent used to analyze the redox status of cultured PBMCs, we next sought to validate the accuracy of the chosen reagent and fluorescence reading system to evaluate PBMC activation in the IMBG. From parallel cultures of healthy donor PBMCs (n = 5), PBMCs were divided into two fractions, which were either (i) stimulated with Dynabeads according to the standard IMBG protocol or (ii) incubated for 2 days in the absence of any stimulus. For the stimulated and unstimulated fractions, five PBMC mixtures, each containing the same total number of cells, were generated at the following proportions: Condition 1, 0% stimulated cells + 100% unstimulated cells; Condition 2, 25% stimulated cells + 75% unstimulated cells; Condition 3, 50% stimulated cells + 50% unstimulated cells; Condition 4, 75% stimulated cells + 25% unstimulated cells; Condition 5, 100% stimulated cells + 0% unstimulated cells.

**Figure 2:**
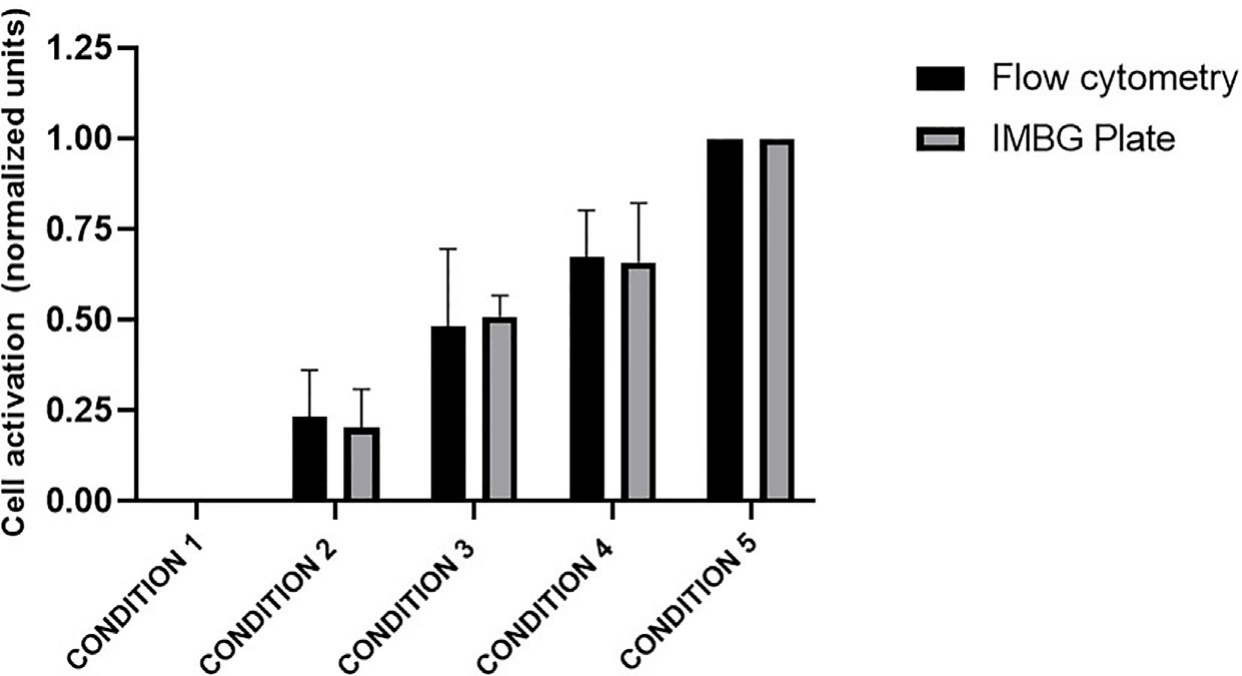
Evaluation of PBMC activation: comparison of the Immunobiogram assay with flow cytometry analysis. Cell activation measured by flow cytometry (black bars) and with resazurin (gray bars), the redox indicator used in the IMBG assay in samples from five healthy blood donors. Cell activation is expressed as normalized fluorescence units and represented in the y axis. Fluorescence values obtained for each of the conditions tested are normalized to those obtained for conditions 1 (100% unstimulated cells) and 5 (100% stimulated cells). The x axis represents the different cells conditions tested: Condition 1, 0% stimulated cells + 100% unstimulated cells; Condition 2, 25% stimulated cells + 75% unstimulated cells; Condition 3, 50% stimulated cells + 50% unstimulated cells; Condition 4, 75% stimulated cells + 25% unstimulated cells; Condition 5, 100% stimulated cells + 0% unstimulated cells. Bar graphs represent mean (bar) and standard deviation (error bars).

The PBMC mixtures were analyzed by flow cytometry using a resazurin reagent distinct from that used in the IMBG, this resazurin is previously described. In parallel, these same mixtures were arranged in IMBG plates using the usual cell concentrations and analyzed following the IMBG protocol, in the absence of immunosuppressant discs (**Figure 2**). In both experiments the fluorescence readings were normalized to those obtained for condition 1 (100% unstimulated cells) and condition 5 (100% stimulated cells). For each condition, similar fluorescence readings were obtained using the two techniques: in both cases the standardized fluorescence signal was reflective of the proportion of activated cells in each condition. An initial Student’s T-test comparing the two sets of measures revealed no significant differences between the two groups. A subsequent two tailed Pearson’s correlation analysis demonstrated a correlation (r = 0.9986, *p* < 0.0001) between the results obtained for the two techniques (IMBG and flow cytometry).

### Data Processing

To generate a normalized representation of the immunosuppressant gradient for each of the tested drugs, we measured the percentage of relative fluorescence units (% RFUs) with respect to the positive control (taken as 100%) and the negative control (taken as 0%). Channel length was normalized to a scale of 0 (the position closest to the immunosuppressant disc) to 100 (the opposite end of the channel).

For each immunosuppressant (IMS) tested, the immunobiogram output consists of 15 immunofluorescence readings taken at sequential points along the concentration gradient in the IMBG channel. This series of fluorescence measurements constitutes a read out of PBMC activation and proliferation across the entire IMS concentration gradient. Fluorescence, expressed as RFUs, was normalized to a scale of 0–100, where 0 and 100 represent the values obtained for the negative control (C-) and positive control (C+), respectively, using the following equation:

[Value (C+, IMS+) – Value (C−, IMS−)]/[Value (C+, IMS−) – Value (C−, IMS−)] X 100

where Value (C+, IMS+) = stimulated immune response in presence of various concentrations (expressed as distance) of IS; Value (C+, IMS-) = Positive control; and Value (C-, IMS-) = Negative control.

**Figure 3:**
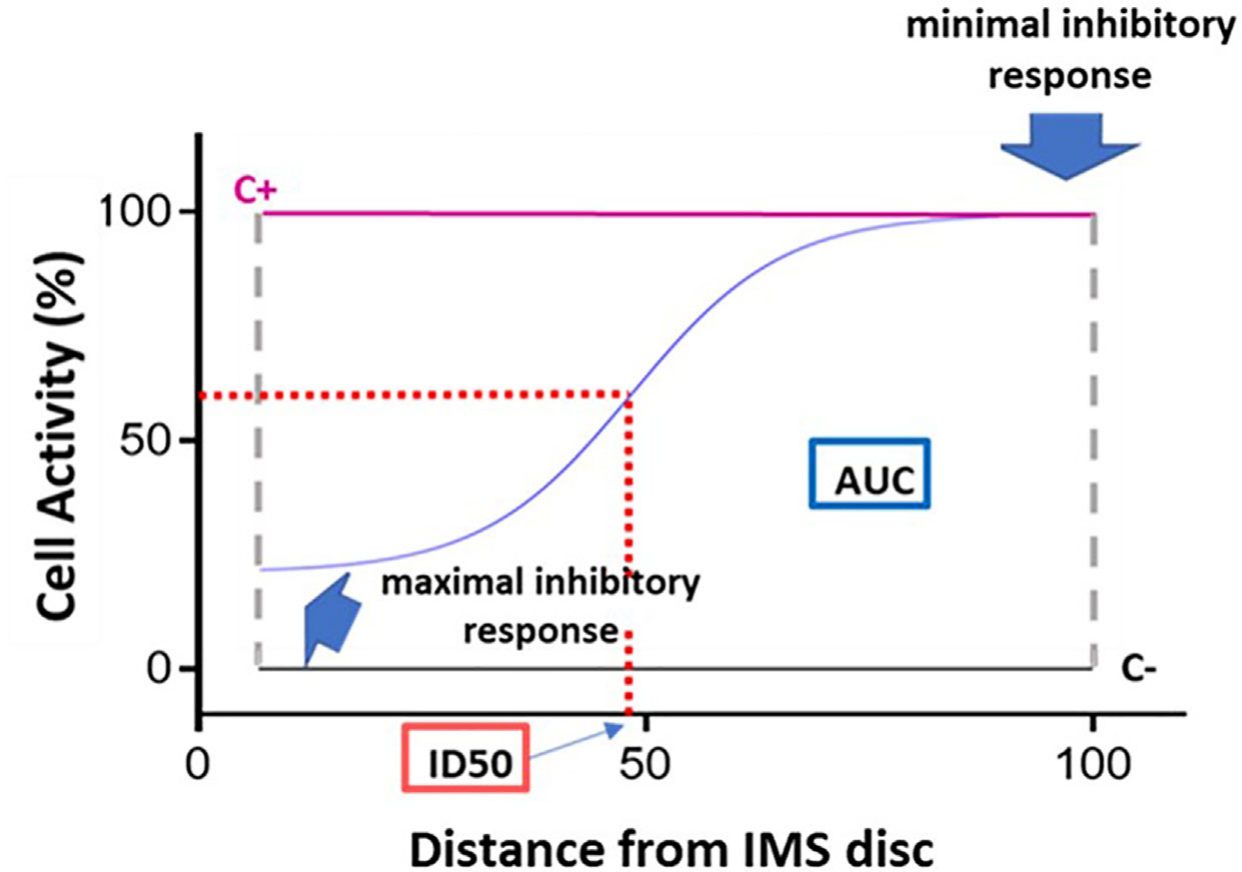
Dose-response curve and key curve parameters. Example of dose-response curve generated based on the 15 fluorescence readings acquired for a given immunosuppressant. The x axis represents distance, normalized to a scale of 0–100, from the point of maximum immunosuppressant (IMS) concentration (0) to the point of minimum IMS concentration (100). The y axis represents cell activity, expressed as RFUs normalized to a scale of 0 (negative control [C-] value) to 100 (positive control [C+]). The following key curve parameters are indicated on the graph: maximal inhibitory response; minimal inhibitory response; ID50 (half maximal inhibitory response); and area under the curve (AUC).

Based on the results obtained, a dose-response curve is generated for each IMS (as shown in **Figure 3**): The x axis represents the IMS concentration gradient, normalized to a scale of 0–100 (0 = point of maximum IMS concentration, closest to the IMS disc; 100 = point of minimum IMS concentration, at the opposite end of the channel), while RFU is plotted on the y axis.

The resulting normalized values were then fitted to the Hill equation, as described in Ritz et al. (28):

$$y=I_{inf}+\;\frac{I_{0}-I_{Inf}}{1+e^{H*\left[log\left(x\right)-log\left(ID50\right)\right]}}$$

The following key curve parameters were obtained (**Figure 3**):

- Maximal inhibitory response (*IRMAX*): PBMC inhibition in the presence of the maximum concentration of the IS (at distance 0). This value corresponds to the fluorescence reading obtained for the first of the 15 points along the IMBG channel (i.e., the first data point on the x axis).
- Minimal inhibitory response *(IRMIN):* PBMC inhibition in the presence of the minimum concentration of the IS. This value is calculated as the mean of the fluorescence readings obtained for the last three points along the IMBG channel (i.e., the last three data points on the x axis).
- Half-maximal inhibitory response *(ID50)*: This parameter is analogous to the pharmacokinetic concept of half-maximal inhibitory concentration (IC_50_), and represents the point on the x axis at which 50% PBMC inhibition is observed. It is calculated as the point on the x axis corresponding to 50% of the y axis value obtained after subtracting the first y axis value from the *IRMAX* value.
- *H* (Hill coefficient): This parameter reflects the slope of the fitted curve.
- Area Under the Curve (AUC): where the y axis represents the extent of PBMC activation as measured on a scale of 0 (minimal activation or maximal immunosuppression) to 100 (maximal activation or minimal immunosuppression). The area under the curve (AUC) corresponds to the level of activation of the PBMCs in the presence of the IS.

### Segmentation Analysis

Using the data obtained from the IMBG assays for each patient, segmentation analyses (quadrant analysis) were performed to classify patients based on their PBMC responses to the battery of immunosuppressants. For the purpose of this analysis we focused on two parameters: AUC and IRMAX; a lower AUC value indicates reduced immune activity in the presence of the IMS (i.e., greater PBMCs sensitivity to the IMS), and a lower IRMAX indicates marked inhibition of cellular activation at maximal IMS concentration (also indicating greater PBMC sensitivity to the IMS).

**Figure 4:**
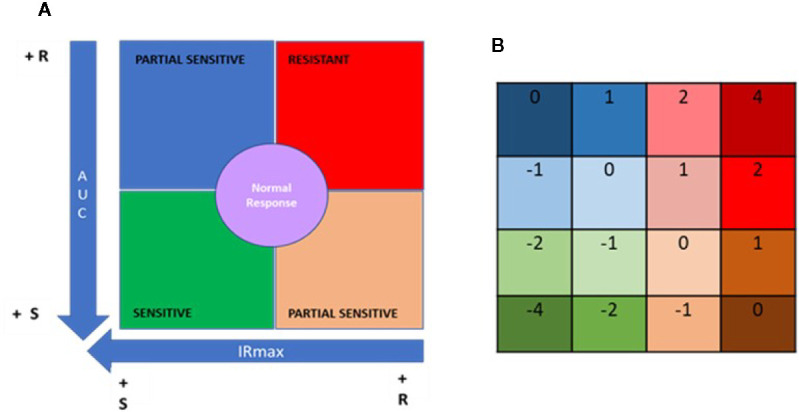
Quadrant analysis to categorize patients based on immunobiogram results. **(A)** For a given immunosuppressant, a resistance map is generated after plotting the maximal inhibitory response (IRMAX) against the area under the curve (AUC) for the entire study population. The graph as then divided into 4 primary quadrants, based on the median IRMAX and AUC values, corresponding to sensitive (green), resistant (red) or partially sensitive (blue and salmon) profiles. The central area of the graph, defined by the 37.5–62.5% percentiles for IRMAX and AUC values, corresponds to a normal response (purple). **(B)** Each of the 4 primary quadrants was further subdivided into 4 subquadrants based on the respective Q1 and Q3 values for AUC and IRMAX, resulting in a resistance map consisting of 16 subquadrants, to which a specific numerical weight was assigned. Based on these values, the patient treatment score was calculated using the formula provided in the *Materials and Methods*.

For each patient these two parameters were plotted against one another (x axis, IRMAX; y axis; AUC) to generate a “resistance map” for the entire study population for each IMS tested. Next, based on the median IRMAX and AUC values for the study population, the resistance map was segmented into four primary quadrants corresponding to discrete IMS sensitivity/resistance profiles (**Figure 4A**).

Data points falling in the lower left quadrant (low IRMAX and low AUC values) correspond to patients sensitive to the IMS tested. Data points falling in the upper right quadrant (high IRMAX and high AUC values) correspond to patients resistant to the IMS tested. Values within this quadrant are closer to those obtained for positive control samples. Data points that fall in either of the remaining quadrants (upper left quadrant: low IRmax and high AUC values; lower right quadrant: high IRMAX and low AUC values) correspond to patients that show only partial sensitivity to the IMS tested.

In addition, a normal response area in the resistance map was defined based on the 37.5% to 62.5% percentiles for each of the plotted parameters. Patients in this area had AUC and IRMAX values close to the respective median values obtained for the study population. To further refine our analysis, each of these four aforementioned quadrants was divided into four subquadrants defined based on the respective Q1 and Q3 values for AUC and IRMAX, resulting in a resistance map divided into 16 subquadrants corresponding to discrete resistance-sensitivity profiles plus the central normal response region.

To interpret the data obtained from the IMBG assay, a patient treatment score was generated based on the resistance maps created for each IMS. The patient treatment score reflects the patient’s sensitivity/resistance profile as determined only for the IMS with which they were being treated when samples were obtained. To calculate this score, a value is assigned to each of the 16 subquadrants (**Figure 4B**); for each IMS prescribed to a given patient, a value is assigned according to the subquadrant in which the corresponding datapoint lies. The patient treatment score is then calculated as the sum of the subquadrant values for each of the IMS prescribed and tested, divided by the total number of IMS prescribed. Given that TAC and MPA are considered pivotal treatments, the subquadrant values obtained for these two drugs were multiplied by two, and an additional unit added to the denominator in cases in which either of these two IMS were included in a patient’s treatment regimen. This is shown in the following formula, which was used to calculate the patient treatment score:

$$\frac{\left(IMS1\right)+\left(IMS2\right)+\ldots \;\left(IMSn\right)}{\left(No.\;of\;IMS\;taken\;by\;the\;patient\right)+1\;\left(if\;MPA\;is\;taken\right)+1\;\left(if\;TAC\;is\;taken\right)}$$

Where n = the total number of IMS taken by the patient.

The final score lies on a quantitative scale (see below) of -4 (highly sensitive) to +4 (highly resistant), where 0 corresponds to a “normal” response.

**Table:** 

Sensitive	Normal response	Resistant
-4 to ≤-0.3	-0.3 to 0.3	≥0.3 to +4

### Statistical Analysis

Statistical analysis was performed using the statistical package GraphPad Prim 8 (version 8.4.2). Clinical and biochemical data and patient treatment scores were analyzed using descriptive statistics. The distribution of the patient treatment score data was assessed using a Kolmogorov-Smirnov normality test, and patient treatment scores were compared by one-way analysis of variance (ANOVA), followed by *post hoc* comparisons using the Tukey test. Significance was established at *p* < 0.05.

## Results

### Healthy Blood Donors

**Figure 5:**
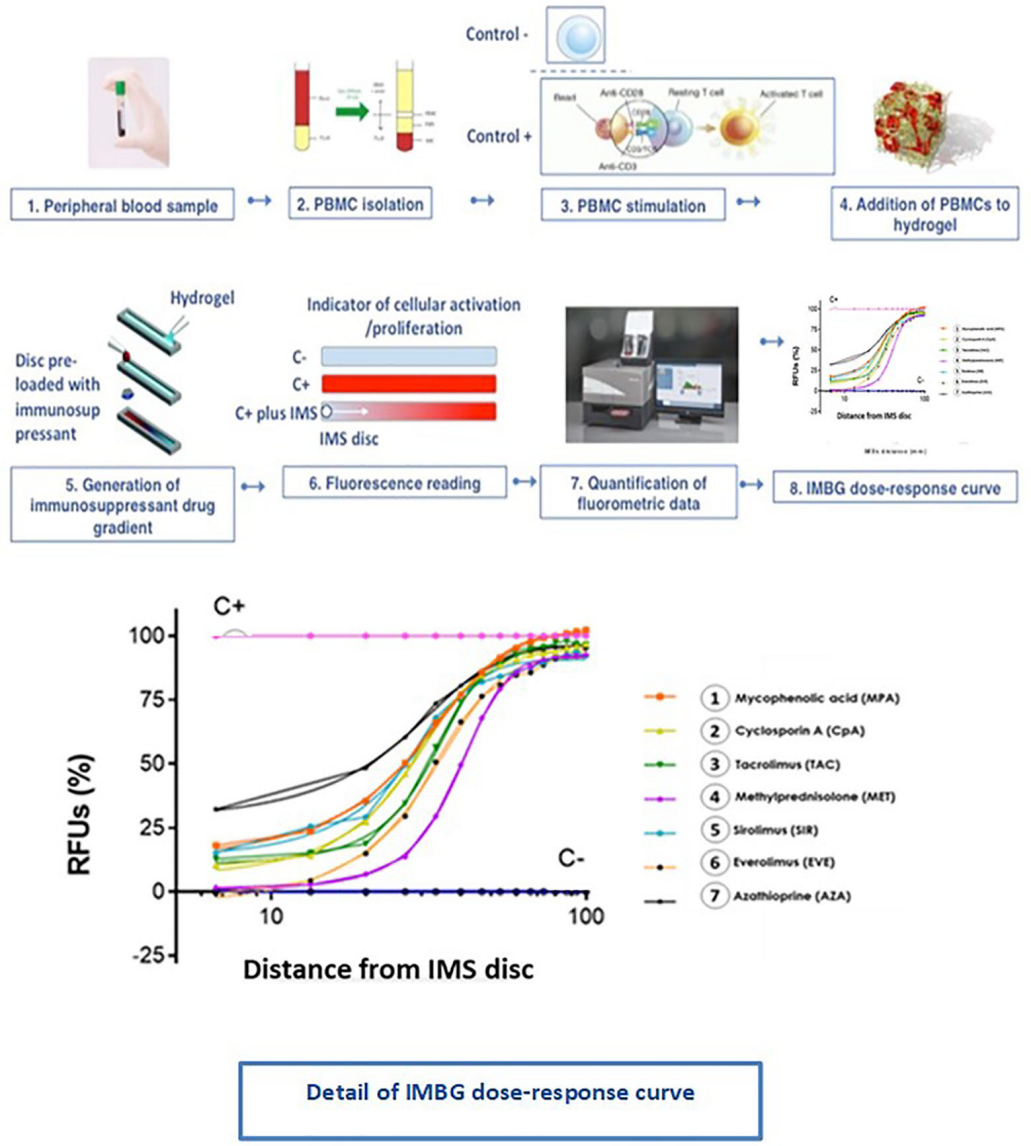
Immunobiogram assay: experimental procedure. PBMCs are extracted from the patient’s blood sample and are immunologically stimulated to induce their activation and proliferation. These activated PBMCs are embedded in a hydrogel substrate, which is then loaded into segregated channels in the IMBG plate. PBMCs in each channel are then exposed to a concentration gradient of a different immunosuppressant, after which PBMCs activation and proliferation along the concentration gradient is measured using a resazurin-based assay, providing a fluorescence read-out of the immune cell response to each immunosuppressant. For each immunosuppressant, dose-response curves are generated based on the 15 immunofluorescence readings taken at sequential points along the concentration gradient in the IMBG channel.

The feasibility of the IMBG method was firstly assessed in a clinical setting using blood samples from 34 apparently healthy adult donors. PBMCs were obtained and assayed following the procedure described in the Materials and Methods (**Figure 5**).

**Figure 6:**
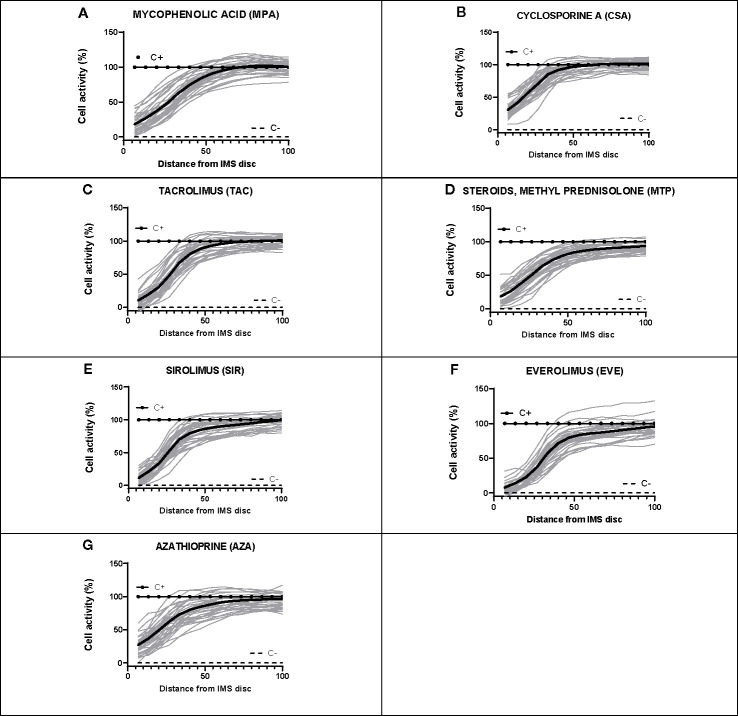
Dose-response curves for each of the seven immunosuppressants tested in healthy blood donor samples. **(A–G)** IMBG curves (in gray) obtained for healthy blood donor samples normalized to positive (C+) and negative controls (C-), as well as the mean curve (in black), obtained for each of the immunosuppressants tested. The x axis represents distance, normalized to a scale of 0–100, from the point of maximum (0) to minimum (100) IMS concentration. The y axis represents cell activity, expressed as RFUs normalized to a scale of 0–100, where 0 and 100 represent the values obtained for the negative (C-) and positive (C+) control, respectively.

For each subject, dose-response curves were generated from IMBG fluorescence data depicting the effect of the seven different immunosuppressants on PBMC proliferation/activation, and corresponding key curve parameters were calculated (data not shown). For all subjects and for each of the IMS tested, the typical dose-response curves obtained from the IMBG are shown in **Figure 6**. For a given IMS, we observed inter-subject differences in the cellular response to a given concentration. This suggests that for each IMS tested, the IMBG provides an individualized read-out of the subject’s sensitivity, which in turn is likely influenced by a variety of factors, both intrinsic and extrinsic (e.g., prior treatment exposure).

### Kidney Transplant Recipients

To further investigate this proposed correlation between immunobiogram results and the subject’s clinical and/or treatment status, we next explored the potential of the IMBG to characterize individual sensitivity to the same battery of immunosuppressant drugs in a population of KT recipients clinically categorized according to their immunological risk of rejection. Clinical, immunological, and sociodemographic characteristics of all patients are shown in **Table 1**.

PBMCs from all patients were isolated from blood samples and assayed using the IMBG. For each patient, dose-response curves were generated, and the corresponding key curve parameters calculated (data not shown). For each patient, we determined sensitivity to the immunosuppressive medication with which they were being treated upon enrolment in the study. The patient treatment scores calculated for all patients are shown in **Table 2**.

**Table 2:** Patient treatment score for each member of the study cohort, stratified according to immunological risk of transplant rejection (low, intermediate, and high risk).

Low risk (n = 21)	Intermediate risk (n = 20)	High risk (n = 19)
-4	-0.66	2.5
-2	-1.5	0.4
-4	0.8	0
-2.5	-1.6	1.2
-2	0.4	0.8
0	0	-1.2
0	-1.6	-0.4
-4	-2.4	-0.8
-2.5	-1.33	1
1.5	2.5	-1.6
-1.5	-0.4	-0.4
-1.5	0	1.8
-4	-0.5	0
0.8	3	0
2	1	-0.5
1.5	0	0.8
1.5	-1.5	1.6
-0.66	-0.55	0
0.66	2.6	1.2
-1	2.25	
-1		

**Figure 7:**
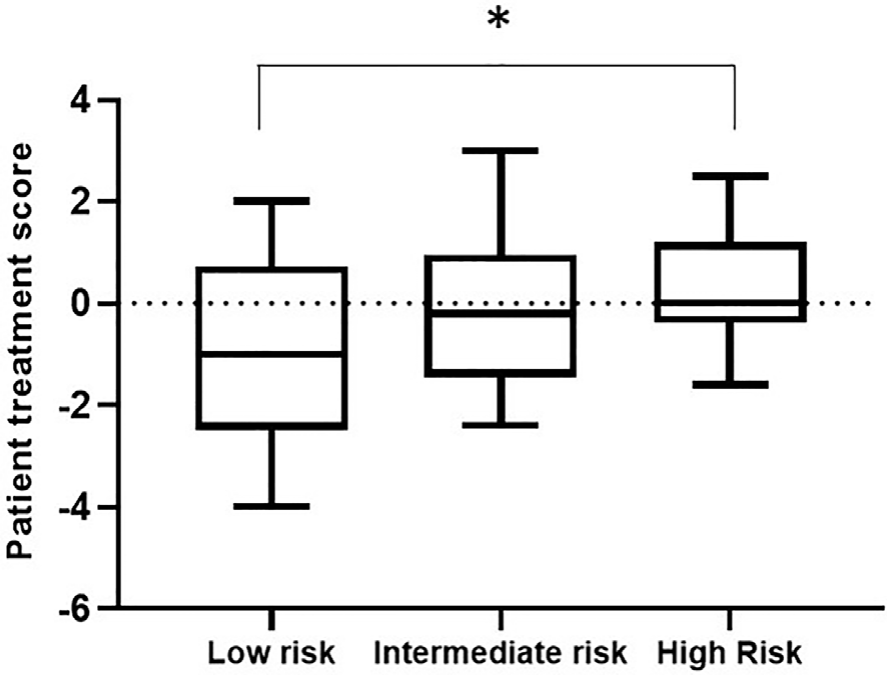
Boxplot depicting patient treatment score according to immunological risk of transplant rejection (low, intermediate, and high risk). Data are expressed as the median and interquartile range. Bars depict the maximum and minimum values of the data series. Patient treatment scores for the low-risk group were significantly lower than those for the high-risk group. *p < 0.05.

Within a given population, a lower patient treatment score indicates greater sensitivity to their prescribed immunosuppressive medication. Descriptive statistics analyses revealed that the mean patient treatment score was lowest in the low-risk group (-1.081), followed by the intermediate-risk group (0.0255), and finally the high-risk group (0.3368) (**Table 3** and **Figure 7**). A Kolmogorov-Smirnov normality test showed that the patient treatment score followed a normal distribution (high-risk: D(19) = 0.1504, p > 0.1000; intermediate-risk: D(20) = 0.1565, p > 0.1000; low-risk: D(21) = 0.1199, p > 0.1000). A one-way ANOVA comparing the mean patient treatment scores of the three groups revealed significant differences between groups (F[2,57] = 4.393, p = 0.0168). *Post hoc* comparisons (Tukey’s test) revealed a significant difference in patient treatment score between the low-risk and high-risk groups (*p* < 0.05), consistent with the fact that these two groups lie at either end of the spectrum of immunological risk.

**Table 3:** Patient treatment score: descriptive statistics.

	Low risk	Intermediate risk	High risk
Number of values	21	20	19
Minimum	-4	-2.4	-1.6
25% Percentile	-2.5	-1.458	-0.4
Median	-1	-0.2	0
75% Percentile	0.73	0.95	1.2
Maximum	2	3	2.5
Range	6	5.4	4.1
Mean	-1.081	0.0255	0.3368
Std. Deviation	1.984	1.575	1.065
Std. Error of Mean	0.4329	0.3523	0.2444

These results indicate that the IMBG test allows quantification of each patient’s sensitivity to their prescribed immunosuppressant medication. Furthermore, we observed an association between patient treatment score and the immunological risk of rejection of the corresponding patient: The mean score of the low immunological risk group was significantly lower than that of the high immunological risk group, indicating greater sensitivity of low immunological risk patients to their prescribed immunosuppressant medication.

## Discussion

Immunosuppressive drugs are widely used for the treatment of several autoimmune diseases and to prevent rejection in organ transplant recipients. However, the clinical efficacy of immunosuppressants varies considerably between individuals, and many patients undergo immunosuppressive therapy despite a poor treatment response and serious adverse effects (19). The driving force behind the immunobiogram assay presented here was to fulfill the unmet clinical need for effective diagnostic tools that help clinicians to tailor immunosuppression therapy to the needs and profile of the individual patient. We have used this assay to simultaneously measure the *in vitro* sensitivity of immune cells from individual patients to a battery of immunosuppressants. The feasibility of the assay was first established by evaluating the response of PBMCs from healthy blood donors to seven different immunosuppressants. Subsequent analysis of PBMCs from KT recipients revealed an association between the patients’ risk of transplant rejection (previously established based on clinical variables) and the corresponding patient treatment score, which reflects sensitivity to the immunosuppressant with which the patient was being treated when blood samples were acquired.

Previous studies have described the use of PBMC functional assays to evaluate the individual pharmacodynamics in patients receiving immunosuppressive therapy, including corticosteroids (29) and other immunosuppressants such as cyclosporine and tacrolimus (30). In these assays, PBMC proliferation and viability can be measured using colorimetric reagents (e.g., the lymphocyte immunosuppressant sensitivity test (LIST) (29, 31) while other authors have used carboxyfluorescein diacetate succinimidyl ester (CFSE)-based flow cytometry for this purpose (32). While these tests have shown potential in predicting the clinical efficacy of immunosuppressants both in immunological disorders and in organ transplant recipients (19), unlike the IMBG they do not enable simultaneous, comprehensive profiling of an individual patient’s sensitivity to a battery of immunosuppressants. This feature of the IMBG means that a single assay can provide clinicians with valuable information on a given patient’s likely response to a range of immunosuppressants, based on which they can evaluate dose adjustments of the patient’s current immunosuppressant regimen or alternative treatment options.

Another unique feature of the IMBG is the use of a 3D hydrogel matrix for PBMC culture. This ensures more accurate simulation of the extracellular matrix scaffold in which T lymphocytes are activated, as well as the target tissues in which they exert their effects (33). The results of several mechano-biological studies have shown that 3D matrix scaffolds of increasing hardness increase the intensity with which T lymphocytes are activated by immunological stimuli (34–36). Furthermore, the 3D environment of the collagen-rich matrix allows passive diffusion of the IMS through the gel along a continuous concentration gradient, in contrast to existing methods that use serial dilutions and conventional culture media (26, 29, 31, 32, 37).

While the aforementioned pharmacodynamic assays use a range of different approaches to trigger lymphocyte activation and expansion, most use non-specific activators, such as phorbol 12-myristate 13-acetate (PMA), phytohemagglutinin (PHA), or different interleukins (mainly IL-2) (38). By contrast, the IMBG uses a canonical antigenic stimulus, activating CD3 and the CD28 coreceptor using Dynabead-conjugated antibodies (24, 39). This dual antigenic activation better simulates the biological process in a clinical setting. Moreover, the surrounding 3D matrix increases the efficiency with which antigenic activation occurs by better mimicking the biomechanical forces involved (40).

We used the IMBG to characterize the sensitivity/resistance profile of individual KT recipients to the immunosuppressant with which they were being treated (i.e., the patient treatment score) when blood samples were taken. Interestingly, we observed an association between this score and the patient’s risk of transplant rejection, as determined based on clinical parameters. Patients with a greater risk of rejection showed reduced sensitivity to their prescribed immunosuppressant, while those with the lowest risk of rejection showed greater sensitivity to their prescribed medication. The IMBG assay thus provides information on the efficacy of the patient’s prescribed immunosuppressant(s), which affects their clinical state and, consequently, their risk of rejection. The patient treatment score therefore constitutes a potential index of the efficacy of the currently prescribed immunosuppressant therapy. Combined with clinical data, the results of the IMBG assay could facilitate the decision-making process when clinicians need to consider changes to a patient’s immunosuppressant treatment regimen.

Future studies using samples acquired before transplantation and in the early post-transplantation phase and involving larger cohorts of kidney transplant recipients with a broader spectrum of immunological risk, will be required to confirm these findings. These and other studies will be essential to validate the present findings and to identify other possible applications of the IMBG assay, including its potential in patients with other conditions that require immunosuppressive therapy.

It should be borne in mind that a patient’s sensitivity to a given immunosuppressant is not a fixed parameter but fluctuates over time in response to various stimuli (41). One factor implicated in this dynamic is the establishment of resistance to a prescribed immunosuppressant. There is evidence that immunosuppressive therapy can upregulate the expression of proteins implicated in cellular mechanisms of drug resistance (42). Repeated IMBG assays conducted serially during treatment could help clinicians to monitor potential changes indicative of the development of resistance, and to determine whether this is specific to the patient in question, and/or is drug-dependent, as previously demonstrated in patients receiving long-term corticosteroid treatment (43).

In conclusion, the fact that the IMBG assay allows characterization of the patient’s sensitivity/resistance to their prescribed immunosuppressant points to a potential role of this test in the personalized optimization and monitoring of immunosuppressive therapy, and in the clinical study of the mechanisms underlying resistance to immunosuppression.

## Data Availability Statement

The raw data supporting the conclusions of this article will be made available by the authors, without undue reservation.

## Ethics Statement

The studies involving human participants were reviewed and approved by Hospital La Paz and by Hospital Puerta de Hierro Ethics and Research Committee. The patients/participants provided their written informed consent to participate in this study.

## Author Contributions

JP and CJ designed the clinical protocol, included the patients, and obtained the clinical samples, reviewed the statistical analyses, and contributed to and reviewed the final draft. LA and DB designed the mathematical algorithms used to analyze the bioassay output, designed the software for final analysis of the bioassay data, reviewed the statistical analyses, and contributed to the final draft. AO-C designed the mathematical algorithms used to analyze the bioassay output and the statistical analysis, participated in the preliminary data analysis, reviewed the statistical analyses, and contributed to the final draft. TD compiled all data for the analysis, designed, led, and reviewed the statistical analyses, and contributed to the final draft. JP contributed to the design of the protocol, reviewed the statistical analyses, and reviewed and contributed to the final draft. IP is coinventor of the cellular assay evaluated, designed the clinical protocol, oversaw the design of the mathematical algorithms used to analyze bioassay outputs, reviewed the statistical analyses, and contributed to the final draft. All authors contributed to the article and approved the submitted version.

## Funding

Research was funded by a competitive grant from the European Commission (SME Instrument – Phase II), Project: TRANSBIO (Id 733248) “Cellular BIOtechnology for prognosis and monitoring in renal TRANSplantation” (https://cordis.europa.eu/project/id/733248).

## Conflict of Interest

This research involved the use of proprietary technologies belonging to BIOHOPE and covered by European Patent: EP 17 382 399.8 “METHOD FOR PREDICTING AND MONITORING CLINICAL RESPONSE TO IMMUNOMODULATORY THERAPY”. Inventors (current or former employees at Biohope): Javier Dotor de las Herrerias, Marianna di Scala, Veronica Sanchez, Isabel Portero Sanchez.

AO, TD, and IP are employees at Biohope. LA and BD were employed by Adamas Engineering Consulting.

The authors declare that this study was sponsored by Biohope. Biohope was beneficiary of a full funding from a competitive grant from the European Commission to conduct the study, and coordinated the study and the assignation of the adjudicated (and by EU audited) funding to the entities (research institutions, CRO and statistical company) that took part in the study.

The remaining authors declare that the research was conducted in the absence of any commercial or financial relationships that could be construed as a potential conflict of interest.
