# Expression of Concern: The immunobiogram, a novel *in vitro* assay to evaluate treatment resistance in patients receiving immunosuppressive therapy

**DOI:** 10.3389/fimmu.2023.1272763

**Published:** 2023-08-15

**Authors:** Frontiers Editorial Office

With this notice, Frontiers states its awareness of concerns regarding the authorship of “The Immunobiogram, a Novel *In Vitro* Assay to Evaluate Treatment Resistance in Patients Receiving Immunosuppressive Therapy” published on 25 January 2021. Specifically, concerns have been raised that three individuals, Veronica Sánchez Rodríguez, Marianna di Scala, and Javier Dotor, have been excluded from the author list. An investigation was conducted in line with COPE guidelines involving the authors’ institution and the funding body, but unfortunately no conclusion could be reached.

This Expression of Concern was approved by the Chief Editors of Frontiers in Immunology and the Chief Executive Editor of Frontiers. The authors do not agree to this Expression of Concern.

